# Mitochondrial stress activates YAP/TAZ through RhoA oxidation to promote liver injury

**DOI:** 10.1038/s41419-024-06448-5

**Published:** 2024-01-15

**Authors:** Ari Kwon, Na Young Lee, Jae-Hyun Yu, Myeung Gi Choi, Jeongwoo Park, Ja Hyun Koo

**Affiliations:** College of Pharmacy and Research Institute of Pharmaceutical Sciences, Seoul National University, Seoul, 08826 Korea

**Keywords:** Mechanisms of disease, Stress signalling, Mitochondria

## Abstract

Yes-associated protein (YAP) and WW domain-containing transcription regulator protein 1 (WWTR1; also known as TAZ) are the main effectors of the Hippo pathway and their dysregulation contributes to diseases in tissues including the liver. Although mitochondria are capable of transmitting signals to change transcriptomic landscape of diseased hepatocytes, such retrograde signaling and the related nuclear machinery are largely unknown. Here, we show that increased YAP activity is associated with mitochondrial stress during liver injury; and this is required for secondary inflammation, promoting hepatocyte death. Mitochondrial stress inducers robustly promoted YAP/TAZ dephosphorylation, nuclear accumulation, and target gene transcription. RNA sequencing revealed that the majority of mitochondrial stress transcripts required YAP/TAZ. Mechanistically, direct oxidation of RhoA by mitochondrial superoxide was responsible for PP2A-mediated YAP/TAZ dephosphorylation providing a novel physiological input for the Hippo pathway. Hepatocyte-specific Yap/Taz ablation suppressed acetaminophen-induced liver injury and blunted transcriptomic changes associated with the pathology. Our observations uncover unappreciated pathway of mitochondrial stress signaling and reveal YAP/TAZ activation as the mechanistic basis for liver injury progression.

## Introduction

Mitochondria are essential organelles in hepatocytes that play a central role in energy homeostasis and xenobiotics metabolism [1]. Mitochondrial stress and dysfunction are commonly associated with various liver pathologies including metabolic dysfunction-associated steatohepatitis and cirrhosis [2, 3]. The contribution of mitochondrial stress in liver injury has been established in human and animal models. For example, N-acetyl-*p*-benzoquinone imine, the toxic metabolite of acetaminophen, primarily targets the mitochondria to cause severe dysfunction and oxidative stress [4–7]. Despite their partial autonomy, mitochondria largely rely on nucleus-encoded proteins and regulatory signals [8]. On the other hand, mitochondria can elicit a retrograde response, which activates the expression of nuclear genes to alter intracellular and extracellular environments to adapt to the conditions that they encounter [9]. Mitochondrial stress can induce pro-inflammatory cytokine production in parenchymal cells and promote immune cell infiltration [10]. Therefore, one can anticipate that mitochondrial stress upon liver injury may cause transcriptomic changes in the nucleus through a retrograde response, thereby inducing secondary inflammation. Nonetheless, it remains unclear how damaged mitochondria control the nuclear machinery and which transcription factor(s) are responsible for such regulation.

The Hippo pathway, which was originally discovered in *Drosophila*, plays an evolutionarily conserved role in the regulation of organ growth, development, and homeostasis [11]. Dysregulation of the Hippo pathway contributes to diverse pathophysiological functions, including macrophage recruitment [12]. YAP and TAZ are the two main effectors of this pathway. In non-dividing cells, phosphorylation of multiple residues by LATS1/2 sequesters YAP/TAZ in the cytosol. LATS1/2 repression through inhibition of upstream kinases or activation of RhoA leads to dephosphorylation, nuclear translocation, and activation of YAP/TAZ, which then initiate target gene transcription through heterodimeric binding with the TEA domain family of transcription factors [11].

In this study, we showed that mitochondrial stress activates YAP/TAZ in damaged hepatocytes. We discovered that mitochondrial stress induced by various inputs activates YAP/TAZ and this significantly contributed to the transcriptomic landscape associated with mitochondrial stress. Mechanistically, direct sensing of mitochondrial superoxide by RhoA was critical. Our study further aimed to focus on the mechanistic insights into the regulation of YAP/TAZ during liver injury and how mitochondria transfer their signals to induce global changes in gene transcription during liver disease progression.

## Results

### YAP activation is associated with mitochondrial stress and inflammation caused upon liver injury

**Fig. 1:**
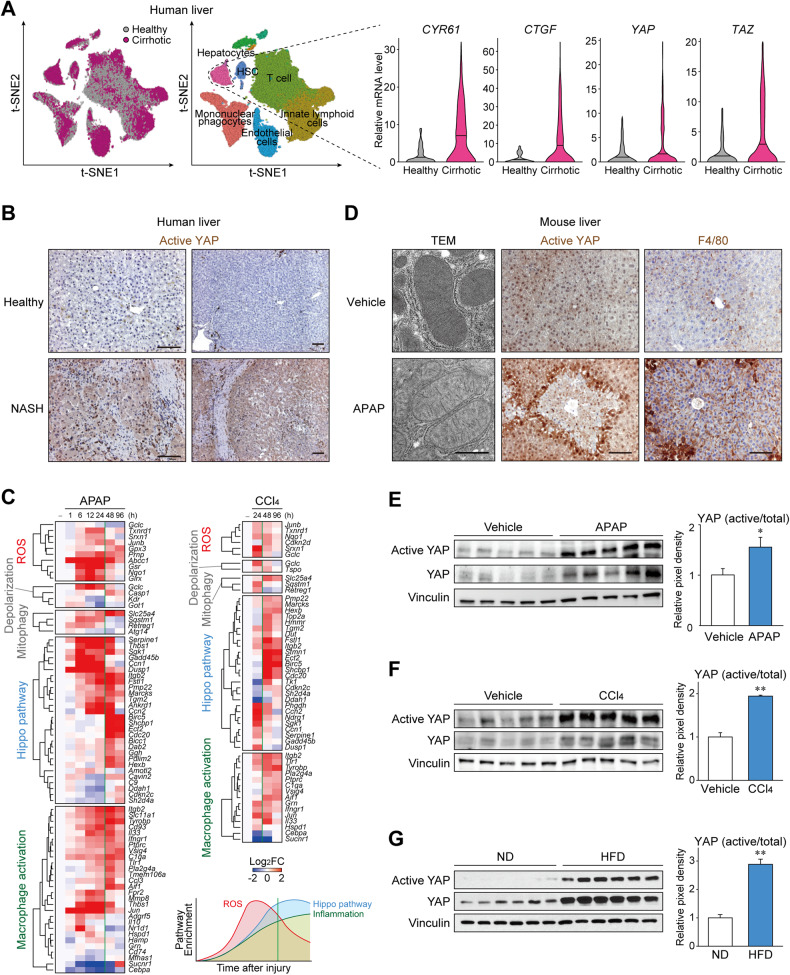
YAP activation is associated with mitochondrial stress and inflammation upon liver injury. **A** *Left*, clustering of single cells by injury condition and cell lineage, from cirrhotic and healthy human livers. *Right*, transcripts from cells with hepatocyte lineage were analyzed for YAP/TAZ and target gene expression. The data were extracted from GEO GSE136103 (*n* = 5 each). **B** Immunostaining for active YAP in liver from a non-alcoholic steatohepatitis patient or a healthy donor. Active YAP antibody recognizes only the non-phosphorylated form. Scale bars: 100 μm. **C** Heatmaps show temporal expression patterns of genes involved in reactive oxygen species (ROS) signaling, mitochondrial membrane depolarization, mitophagy, the Hippo pathway, and macrophage activation pathways, after acetaminophen (APAP) or CCl_4_ intoxication in mouse liver. The data were extracted from GEO GSE167032 and GSE167033. Averages of log2 fold change are shown for each group (*n* = 5). **D** Transmission electron micrograph (left) and immunohistochemistry for active YAP and F4/80 (middle and right) in mouse liver after APAP intoxication. Mice are starved overnight and treated with acetaminophen (300 mg/kg) or vehicle for 24 h. Scale bars: 1 μm (left), 100 μm (middle and right). **E**–**G** Immunoblottings for active YAP in the livers with hepatocyte injury. **E** The mice were sacrificed 24 h after single injection of APAP (300 mg). **F** The mice were sacrificed 24 h after single injection of CCl_4_ (1 ml/kg). **G** Mice fed with 60 kcal% high-fat diet (HFD) or normal diet (ND) for 8 weeks were used. For **E**–**G**, data represent the mean ± SEM. **P < 0.01, by Student’s *t* test.

To identify the clinical relevance of Hippo pathway in the injured liver, we analyzed single-cell RNA sequencing (scRNA-seq) data against liver cells obtained from cirrhotic patients or healthy donors (GSE136103) since reduced mitochondrial potential and increased mitochondrial stress are frequently observed in cirrhotic livers [3]. Single-cell-level analysis allowed us to compare transcriptional changes associated with hepatocyte deterioration in a cell type-specific manner. The expression levels of representative YAP/TAZ target genes, namely *CYR61* and *CTGF*, were significantly upregulated in hepatocytes obtained from cirrhotic livers (Fig. 1A), whereas non-parenchymal cells showed comparable gene expression (Supplementary Fig. 1A). Although YAP/TAZ activity is generally regulated through phosphorylation rather than transcription, expression of *TAZ* per se was also increased. In line with the scRNA-seq data, active YAP levels were particularly higher in hepatocytes from a patient with metabolic dysfunction-associated steatohepatitis with fibrosis, an antecedent pathological condition associated with mitochondrial stress [2], when compared to those from a healthy individual (Fig. 1B).

To confirm the involvement of Hippo pathway in liver injury, bulk RNA sequencing (RNA-seq) data from animal models of acute liver injury induced using APAP were analyzed (GSE166868). Notably, the temporal pattern of RNA expression showed sequential changes in reactive oxygen species (ROS) signaling, Hippo pathway, and macrophage activation signaling upon APAP administration (Fig. 1C and Supplementary Table 1). Changes in the ROS signaling pathway were observed as early as 1 h after the administration of APAP and lasted for up to 24 h. This was associated with other mitochondrial dysfunction genes such as those of mitochondrial membrane depolarization and mitophagy. The Hippo pathway was changed at a similar onset but the changes persisted for more than 48 h. This was followed by the macrophage activation pathway, which showed relatively slow induction and long duration, which is in line with the clinical observations that APAP overdosing involves a secondary inflammatory response promoted by the release of inflammatory mediators [13, 14]. Another liver injury model using CCl_4_ (GSE167033), which also involves mitochondrial damage [15, 16], showed similar results (Supplementary Table 2). These support the notion that the Hippo pathway regulation is commonly involved in conditions where hepatocytes are exposed to excessive mitochondrial stress and inflammation.

Next, we sought to directly measure the YAP activity in mouse liver. Transmission electron microscopy confirmed severe mitochondrial damage in hepatocytes after APAP injection, as indicated by the disruption of the inner structure and decreased electron density (Fig. 1D and Supplementary Fig. 1B, C). YAP activity was determined by immunostaining using an active YAP antibody, which specifically detects dephosphorylated nuclear YAP. The injured mouse liver showed a dramatic increase in active YAP levels, particularly in the most reactive hepatocytes adjacent to the necrotic area. As expected, the level of macrophage marker F4/80 was also increased, supporting the notion that YAP/TAZ activation coincides with mitochondrial damage and inflammation. Moreover, a further experiment revealed that mitochondrial damage (6 h) precedes YAP activation (12 h) in the liver after APAP, reflecting the sequential changes observed by transcriptomic analysis (Supplementary Fig. 1D). Semi-quantitative comparison by western blotting of whole liver lysates confirmed significant induction of YAP activity upon APAP treatment (Fig. 1E). In addition, a single injection of CCl_4_ was sufficient to considerably enhance the levels of active YAP as well as its representative target genes (Fig. 1F). YAP activity was also increased in a steatohepatitis model induced by high-fat feeding in mice (Fig. 1G). These results revealed that inhibition of the Hippo pathway is a feature of liver injury that are associated with mitochondrial stress.

### Mitochondrial stress activates YAP

**Fig. 2:**
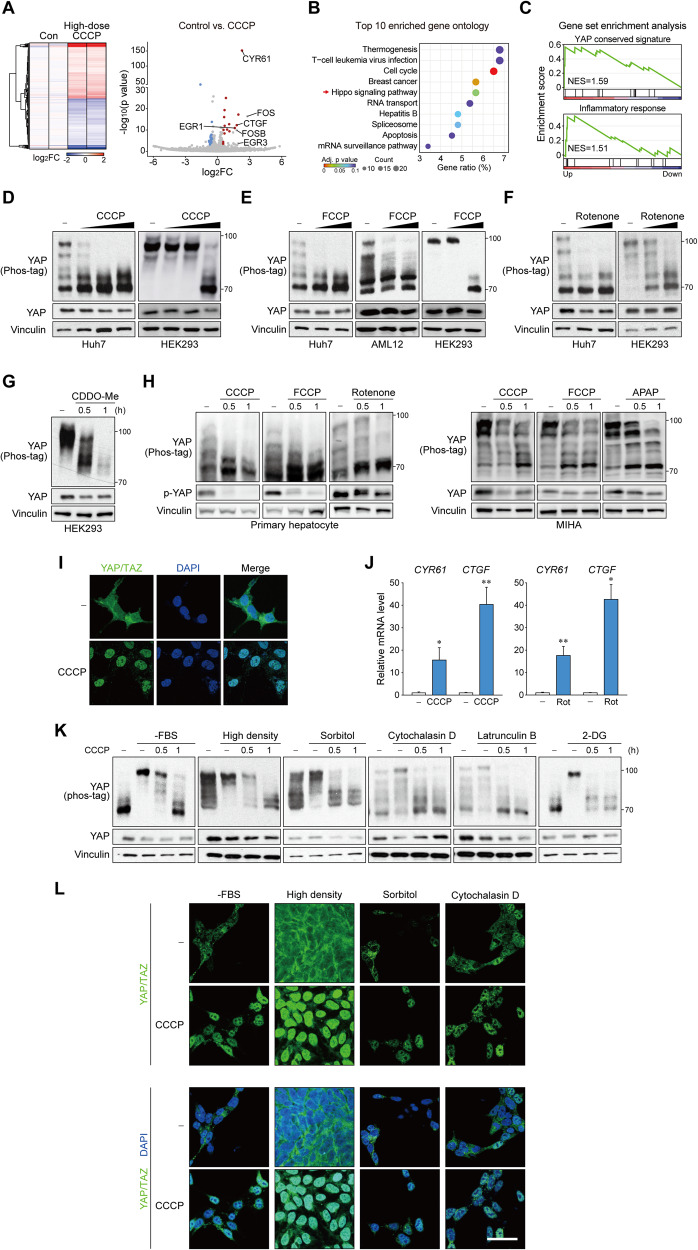
Mitochondrial stress activates YAP. **A** RNA-seq analysis of HEK293 cells treated with CCCP (20 μM) for 3 h. Heatmap of global gene expression (left) and volcano plot of fold change and p value (*right*) is shown. Data points for YAP/TAZ target genes are labeled. Red, upregulated; blue, downregulated. **B** Gene ontology analysis for differentially expressed genes shows the Hippo pathway as a highly enriched KEGG pathway. **C** Gene set enrichment analysis of differentially expressed genes shows enriched YAP/TAZ target gene signature and inflammatory response upon mitochondrial stress. YAP is dephosphorylated by mitochondrial stress. Immunoblots for YAP on Phos-tag or regular gels are shown using samples from different cell lines. Cells were treated with increasing concentrations of CCCP (5, 10, 20 μM) (**D**), FCCP (5, 10, 20 μM) (**E**), Rotenone (1, 2 μM) (**F**) for 1 h. CDDO-Me (2.5 μM) was given for indicated times (**G**). **H** Mitochondrial stress activates YAP in normal hepatocytes. MIHA immortalized human hepatocytes (left) and mouse primary hepatocytes (right) were subjected to mitochondrial stress by CCCP (50 μM), FCCP (20 μM), acetaminophen (50 mM) or rotenone (2 μM). **I** Mitochondrial stress induces YAP/TAZ nuclear localization. HEK293 cells were treated with CCCP (20 μM) for 1 h and then stained for immunofluorescence. Scale bar: 50 μm. **J** YAP/TAZ target genes are upregulated by mitochondrial stress. RT-qPCR analysis were done using HEK293 cells were treated with CCCP (50 μM) or rotenone (2 μM) for 3 h. The data represent the mean ± SEM. *P < 0.05 and **P < 0.01, by Student’s *t*-test. **K**, **L** Mitochondrial stress overrides other Hippo activating signals to induce YAP phosphorylation. HEK293 cells were pretreated with YAP inhibitory signals (see “Methods”), then treated with CCCP (50 μM) for indicated times. Dephosphorylation was determined by Phos-tag gel analyses (**K**) and nuclear localization was visualized by fluorescent immunostaining for YAP/TAZ (**L**). Scale bar: 50 μm. For **A**–**J**, cells were serum-starved for 12 h to 16 h before experiment.

We used carbonyl cyanide 3-chlorophenylhydrazone (CCCP) to identify the causal relationship between mitochondrial stress and YAP. It is widely utilized as a potent inducer of mitochondrial stress at high doses (>5 μM) by promoting superoxide production, structural fragmentation, and an integrated stress response (ISR) [17, 18], while also used as a mitochondrial ionophore at low doses (<2 μM). We performed RNA-seq using cells treated with a high-dose of CCCP (20 μM). Surprisingly, from 914 DEGs (469 upregulated and 445 downregulated genes), YAP/TAZ target genes such as *CYR61*, *CTGF*, *FOS*, *FOSB*, *EGR1*, and *EGR3* were among the top hits (Fig. 2A and Supplementary Table 3). Statistically, the Hippo pathway was a highly enriched KEGG signaling pathway among the DEGs (Fig. 2B). Gene set enrichment analysis (GSEA) further confirmed highly enriched YAP target and inflammation signatures (Fig. 2C).

YAP is inhibited by phosphorylation of multiple residues, which can be observed as mobility shifts on a Phos-tag gel [19]. Treatment with CCCP caused rapid and complete dephosphorylation of YAP and TAZ within 1 h in multiple cell lines (Fig. 2D and Supplementary Fig. 2A). The results were reproduced upon the treatment with carbonyl cyanide 4-(trifluoromethoxy)phenylhydrazone (FCCP)(Fig. 2E). Other mitochondrial stress inducers with different mechanisms, such as rotenone (mitochondrial complex 1 inhibitor) and 2-cyano-3,12-dioxo-oleana-1,9(11)-dien-28-oic acid methyl ester (CDDO-Me; mitochondrial proteolysis inhibitor), also induced YAP dephosphorylation (Fig. 2F, G). YAP activation by mitochondrial stress was also observed in mouse primary hepatocytes and immortalized non-neoplastic hepatocytes, as shown by mobility shifts in Phos-tag gels and a decrease in Ser 127 phosphorylation (Fig. 2H). LATS-dependent phosphorylation of YAP/TAZ results in 14-3-3 binding and cytoplasmic localization [20]. As expected, immunofluorescence staining revealed that CCCP induced nuclear accumulation of YAP/TAZ (Fig. 2I and Supplementary Fig. 2B). Consistent with YAP/TAZ dephosphorylation and nuclear translocation, mitochondrial stress significantly increased the expression of YAP/TAZ target genes as assessed by qRT-PCR (Fig. 2J). Hepatic non-parenchymal cells, namely hepatic stellate cells and Kupffer cells were also able to induce YAP after CCCP treatment (Supplementary Fig. 2C). However, it is noteworthy that YAP was less efficiently activated in these cells. Thus, the signaling pathway may be most physiologically important for homeostasis in hepatocytes.

Considering the robustness of YAP activation, we investigated whether mitochondrial stress could activate YAP under other inhibitory conditions. As a result, CCCP could override all inhibitory signals tested, including serum starvation, high density, osmotic stress by sorbitol, F-actin disruption by latrunculin B or cytochalasin D, and energy stress by 2-deoxyglucose (2-DG) in the aspects of YAP dephosphorylation (Fig. 2K) as well as YAP/TAZ nuclear translocation (Fig. 2L and Supplementary Fig. 2D). These observations demonstrated that mitochondrial stress universally induces rapid and dramatic YAP activation, and possibly regulates the Hippo pathway differently from the canonical kinase cascade.

### MST and MAP4Ks are not the key mediators resulting from mitochondrial stress for YAP activation

**Fig. 3:**
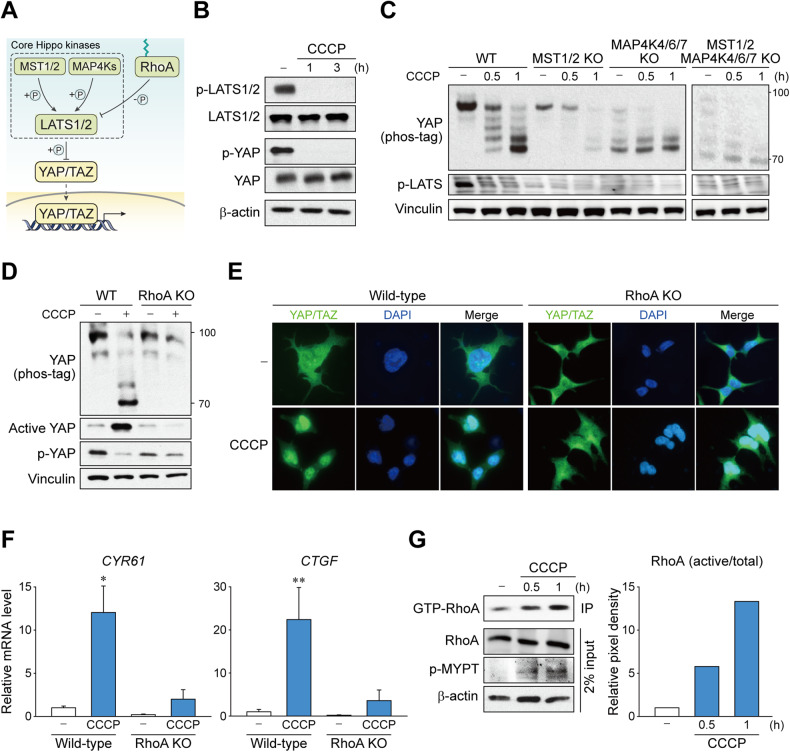
Mitochondrial stress signal through RhoA, but not MST1/2 and MAP4K to activate YAP. **A** Scheme depicting classical Hippo kinase cascade. While LATS1/2 is largely responsible for YAP/TAZ phosphorylation, MST1/2 and MAP4Ks are on the upstream as the core Hippo kinases. **B** Mitochondrial stress induces LATS1/2 inhibition. HEK293 cells were treated with CCCP for indicated times. Phosphorylation (Thr 1079) of LATS1/2 kinases were determined by immunoblotting. **C** Deletion of MST1/2 or MAP4K4/6/7 does not affect mitochondrial stress-induced YAP activation. MST1/2 KO, MAP4K4/6/7 KO, MST1/2-MAP4K4/6/7 KO, and wild-type cells were treated CCCP for indicated times. The phosphorylation of YAP was analyzed by immunoblot using Phos-tag gels. **D**–**F** Mitochondrial stress requires RhoA to induce YAP activation. RhoA KO and wild-type cells were treated with CCCP for 1 h. **D** The phosphorylation of YAP was analyzed by immunoblot using Phos-tag gels. **E** Nuclear localization was determined by fluorescence immunostaining. **F** YAP/TAZ target gene expression was determined by RT-qPCR. The data represent the mean ± SEM. *P < 0.05 and **P < 0.01, by Student’s *t* test. **G** RhoA is activated upon mitochondrial stress. Active RhoA on cells treated with CCCP for indicated times was first immunoprecipitated using antibody specific to GTP-bound form then was immunoblotted for total RhoA. For **B**–**G**, HEK293 cells were serum-starved for 12 to 16 h prior to CCCP (50 μM) treatment.

The key components of the Hippo pathway include LATS1/2, which are the direct upstream factors that phosphorylate and repress YAP/TAZ, and MST1/2 and MAP4Ks, which phosphorylate and activate LATS1/2 (Fig. 3A) [21]. We observed that CCCP strongly repressed LATS1/2 phosphorylation in the hydrophobic motif (Thr^1079^ for LATS1 and Thr^1041^ for LATS2), which indicates the inactivation of LATS1/2 (Fig. 3B). Therefore, we next investigated whether the upstream kinases of LATS1/2 are involved in mitochondrial stress-induced YAP regulation by comparing cell lines with deleted MST1/2 and/or MAP4K4/6/7. However, YAP activation by CCCP was not blocked in MST1/2 KO or MAP4K4/6/7 cells (Fig. 3C). Moreover, YAP dephosphorylation was still observable in cells lacking both MST1/2 and MAP4K4/6/7 altogether. These results indicated that neither MST1/2 nor MAP4Ks are strictly required for mitochondrial stress-induced YAP regulation.

### RhoA is primarily responsible for YAP activation by mitochondrial stress

Several known upstream regulators of LATS were tested, and we identified that RhoA was primarily responsible for YAP activation by mitochondrial stress. CCCP-induced YAP dephosphorylation was completely blocked in RhoA KO cells, which was confirmed by mobility shift using Phos-tag gels as well as active YAP and phospho-YAP immunoblots (Fig. 3D). Consistently, nuclear accumulation and transcriptional activation of YAP/TAZ by CCCP did not occur in the absence of RhoA (Fig. 3E, F). Supporting these results, RhoA activity was augmented by CCCP treatment, as evidenced by the increase in GTP-bound RhoA levels and phosphorylation of the downstream effector MYPT1 (Fig. 3G).

### Mitochondrial superoxide triggers RhoA oxidation for YAP dephosphorylation

**Fig. 4:**
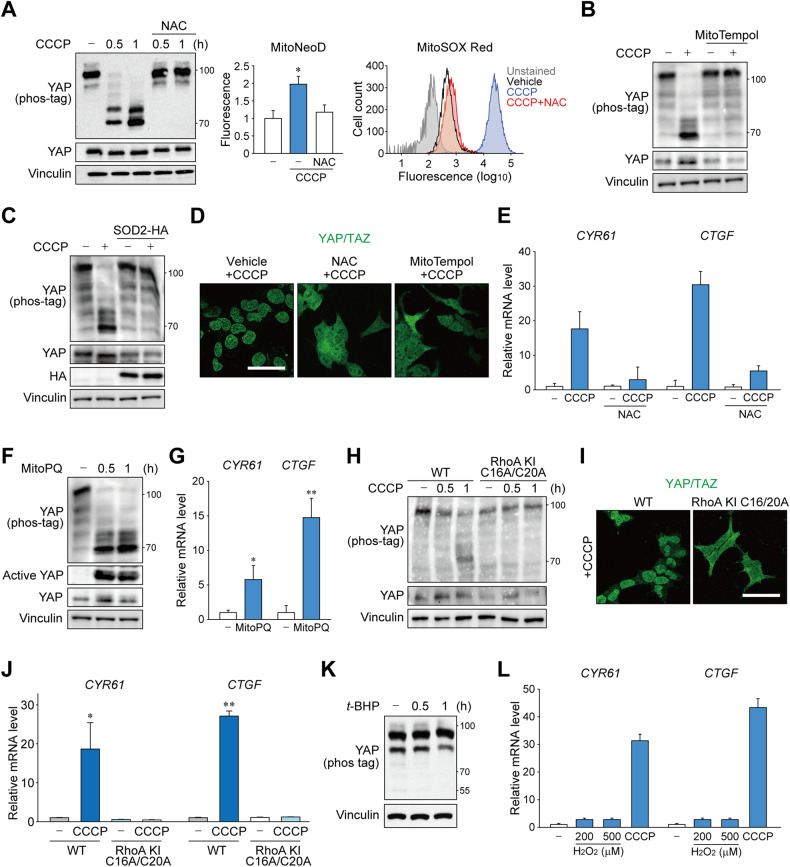
Mitochondrial superoxide triggers RhoA activation for YAP dephosphorylation. **A** Mitochondrial superoxide is essential for CCCP-induced YAP activation. Cells were pretreated with 2 mM N-acetylcysteine (NAC) before CCCP (1 h) treatment and analyzed by immunoblotting (*left*), fluorometry after staining with 5 μM MitoNeoD (middle), or flow cytometry after labeling with 2 μM MitoSOX Red (right). **B** Cells were treated with CCCP (1 h) after mitoTempol (100 μM, 1 h) or vehicle pretreatment, then danalyzed using Phos-tag immunoblot. **C** Phos-tag and regular immunoblots for YAP. Cells were transfected with HA-tagged SOD2 expression vector and treated with CCCP (1 h) after 24 h. **D** Superoxide scavengers impede YAP/TAZ nuclear localization. Cells were treated with CCCP (1 h) after NAC (2 mM), mitoTempol (100 μM) or vehicle treatment, then used for YAP/TAZ immunostaining. Scale bar: 50 μm. **E** qRT-PCR for YAP/TAZ target genes. Cells were treated with CCCP (1 h) after NAC (2 mM) or vehicle treatment. **F**, **G** Phos-tag and regular immunoblots (**F**) and qRT-PCR of target genes for YAP activity assay. Cells were treated with mitoPQ (10 μM) for indicated times or 3 h, respectively. **H**–**J** Cells with superoxide-irresponsible mutant RhoA does not induce YAP activation. YAP phosphorylation was compared in wild-type cells or cells with C16A/C20A knock-in mutation on endogenous RhoA. **H** Phos-tag immunoblots for YAP using cells treated with CCCP for indicated times. **I** Immunostaining for YAP/TAZ after CCCP for 3 h. **J** RT-qPCR analysis for YAP target genes after treatment with CCCP for 3 h. **K** *tert*-butylhydroperoxide (*t*-BHP; 500 μM) were given to cells for indicated times and analyzed using Phos-tag immunoblotting for YAP. (**L**) Cells were treated with increasing concentrations of hydrogen peroxide as indicated or CCCP for 3 h and analyzed by qRT-PCR. For **A**–**L**, HEK293 cells were serum-starved for 12 h to 16 h prior to CCCP (50 μM) treatment.

To further delineate the mechanistic basis of mitochondrial stress-mediated RhoA regulation, we screened a series of mitochondrial dysfunctions that may have been caused by high-dose CCCP. Intriguingly, we observed that elimination of reactive oxygen species by N-acetylcysteine, the first-line treatment option for APAP-induced acute liver injury, completely blocked CCCP-induced YAP activation (Fig. 4A). Scavenging superoxide by pretreatment with a mitochondria-targeted antioxidant mitoTempol or overexpression of mitochondrial superoxide dismutase SOD2 also had similar effect (Fig. 4B, C, Supplementary Fig. 2E, F). Consistently, the antioxidants prevented YAP/TAZ nuclear translocation and target gene induction by CCCP (Fig. 4D, E). The critical role of superoxide production in YAP activation was further confirmed by using a mitochondria-targeted superoxide generator MitoPQ, which dephosphorylated YAP/TAZ and induced its target gene expression (Fig. 4F, G, Supplementary Fig. 2G).

Unlike other GTPases, RhoA has a redox-sensitive motif that directly interacts with superoxide anion radicals [22]. Oxidation of cysteine residues, particularly Cys 20 located adjacent to the GDP-binding site, enhances nucleotide exchange, leading to spontaneous activation of RhoA in cells [23]. We generated a knock-in cell line against endogenous RhoA with two superoxide-sensing cysteine residues mutated to alanine (C16A/C20A)(Supplementary Fig. 3A). Surprisingly, the cells with superoxide-insensitive RhoA were unable to dephosphorylate YAP, prevent nuclear translocation, or induce target gene transcription in response to CCCP treatment (Fig. 4H–J). It is also noteworthy that hydrogen peroxide or *tert*-butylhydroperoxide failed to activate YAP (Fig. 4K, L, Supplementary Fig. 3B), which is in line with the fact that RhoA is only sensitive to superoxide [22]. Mitochondrial dysfunction by CCCP may trigger other signaling pathways such as ISR, mitochondrial proteostasis, and mitophagy (Supplementary Fig. 4A). Recent studies have revealed that various mitochondrial dysfunctions converge on the OMA1-mediated response termed ISR [17, 18]. However, YAP activity was minimally affected by blockade of the ISR pathway achieved using eIF2α S51A knock-in, ATF4 KO, or chemical inhibitor ISRIB (Supplementary Fig. 4B, C). Likewise, inhibition of mitophagy by Drp1 inhibition did not prevent YAP activation (Supplementary Fig. 4D). Impairment of mitochondrial protein import by GFER inhibition was independent of YAP activity (Supplementary Fig. 4E). Ferroptosis was also ruled out since its inhibitor Ferrostatin-1 did not block YAP activation (Supplementary Fig. 4F). Given that superoxide anions are rapidly converted to hydrogen peroxide under physiological settings, it is conceivable that YAP activation occurs when superoxide is produced in excess amounts that cannot be promptly quenched and released through mitochondrial membrane pores or ruptures, such as during acute liver injury. Indeed, antioxidant treatment alone could not inhibit basal levels of YAP activity (Supplementary Fig. 5).

### Mitochondrial stress induces both Lats-dependent and Lats-independent YAP activation

**Fig. 5:**
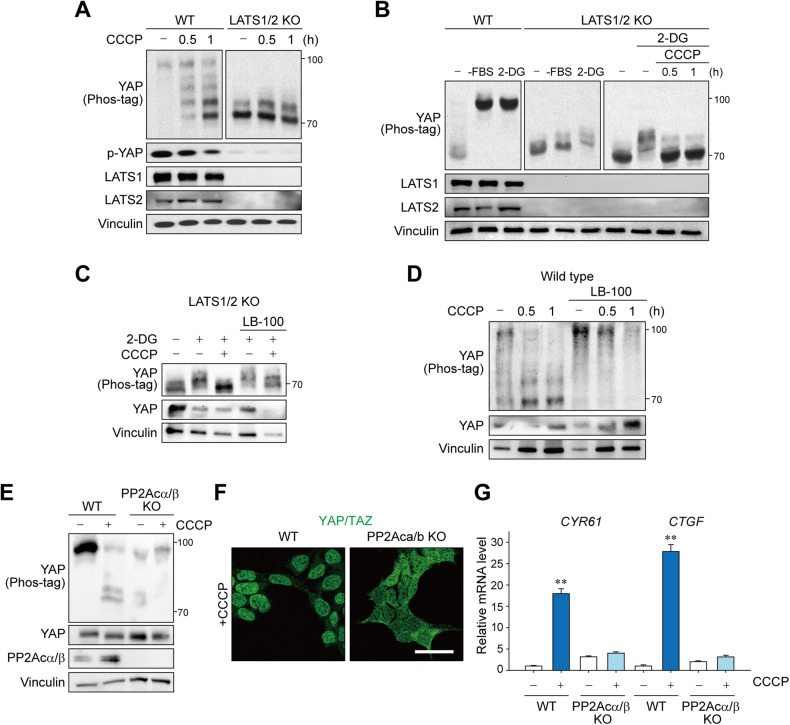
Mitochondrial stress induces YAP dephosphorylation via both LATS-dependent and LATS-independent mechanisms. **A** LATS1/2 are primarily responsible for YAP phosphorylation. LATS1/2 KO and wild-type cells were serum-starved for 12 h, then treated with CCCP, analyzed by immunoblotting. **B** Mitochondrial stress is capable of activating YAP without LATS1/2. Cells were subjected to serum starvation or 2-DG (25 mM) treatment for 1 h, then treated with CCCP, followed by YAP immunoblotting analysis. **C** PP2A inhibition blocks LATS1/2-independent YAP activation. LATS1/2 KO cells were pretreated with 2-DG (25 mM) and LATS1/2 inhibitor LB-100 (5 μM) for 1 h and then treated with CCCP for 1 h. **D** Cells were serum-starved for 16 h, pretreated with LB-100 (5 μM), and given CCCP before immunoblotting. PP2Acα/β KO and wild-type cells were serum-starved for 12 h and subjected to CCCP treatment (1 or 3 h) for YAP immunoblotting (**E**), YAP/TAZ immunostaining (**F**), and qRT-PCR for the target genes (**G**). Scale bar: 50 μm. For **A**–**G**, HEK293 cells and 50 μM CCCP were used.

Lats is the main kinase responsible for YAP/TAZ inhibition [21]. Considering that LATS1/2 were rapidly and robustly dephosphorylated upon CCCP treatment, it is apparent that their inhibition leads to the derepression of YAP/TAZ. Hence, we next examined YAP phosphorylation in LATS1/2 KO cells to confirm whether mitochondrial stress signaling depends solely on LATS1/2. As expected, ablation of LATS1/2 abolished most of the YAP phosphorylation (Fig. 5A), which is in line with the current understanding that LATS1/2 are the major YAP/TAZ kinases. In contrast, energy stress induced by 2-DG induced YAP phosphorylation in LATS1/2 KO cells to some extent (Fig. 5B), which is consistent with a previous report that AMP-activated protein kinase (AMPK) could directly phosphorylate YAP [24]. CCCP was still able to reverse LATS-independent YAP phosphorylation as well, thereby raising the possibility that mitochondrial stress signals also act at a step more proximal to YAP in addition to LATS1/2 inhibition.

PP2A has recently been implicated in mediating RhoA-mediated LATS1/2 inhibition through the STRIPAK complex [25], and it can also directly dephosphorylate YAP [26]. Treatment with LB-100, a PP2A-specific inhibitor, significantly diminished LATS-independent YAP dephosphorylation in LATS1/2 KO cells (Fig. 5C). Moreover, LB-100 inhibited CCCP-induced YAP dephosphorylation in wild-type cells, suggesting that PP2A also serves as a downstream signal mediator of RhoA-mediated, Lats-dependent or -independent signaling (Fig. 5D). Consistently, YAP activation by CCCP did not occur when the two catalytic subunits of PP2A (PP2Acα/β) were deleted, confirming the role of direct dephosphorylation (Fig. 5E–G).

### YAP/TAZ induce mitochondrial stress transcriptome

**Fig. 6:**
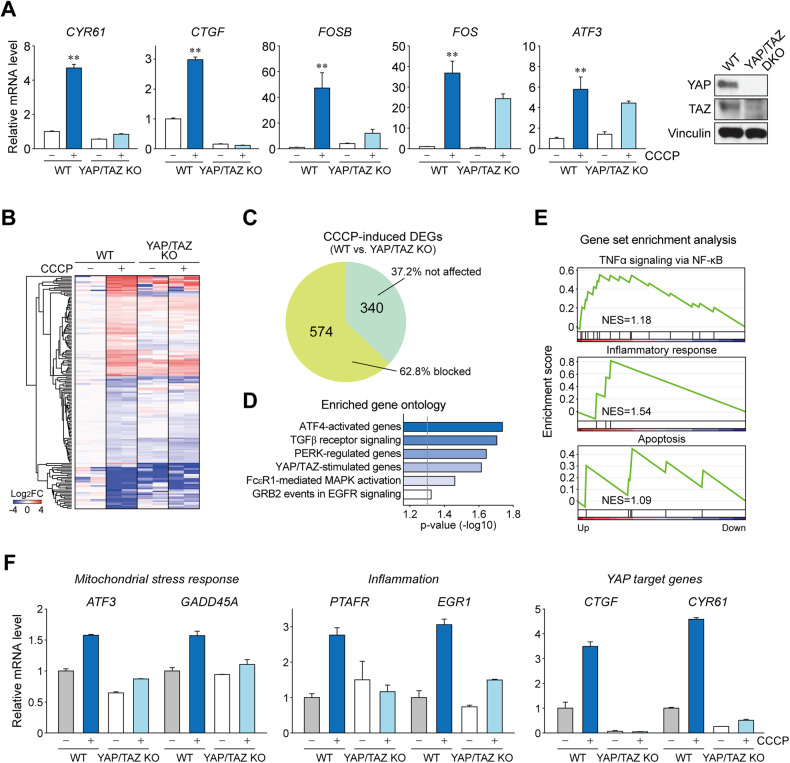
YAP/TAZ induce the mitochondrial stress transcriptome. **A** qRT-PCR analysis for YAP/TAZ target genes were performed using YAP/TAZ KO and wild-type (WT) after CCCP treatment (*n* = 3 per group). **B** RNA-seq analysis of YAP/TAZ KO and wild-type cells was performed and heatmap of CCCP-induced DEGs which are affected by YAP/TAZ KO is shown (*n* = 2 per group). Cells were harvested 3 h after CCCP (20 μM) treatment. **C** Pie chart showing the portion of CCCP-induced DEGs blocked by YAP/TAZ KO. **D** Gene ontology analysis for YAP/TAZ-dependent DEGs. The genes were categorized according to the KEGG pathway. **E** Gene set enrichment analysis of the DEGs shows enriched TNFα, inflammation, and apoptosis signaling pathways, respectively. DEGs were grouped according to the Hallmark gene sets in molecular signature database (MSigDB). **F** Expression of genes related to mitochondrial stress response and inflammation and YAP/TAZ transcriptional targets from the RNA-seq experiment.

Next, we examined whether YAP/TAZ are required for CCCP-induced gene transcription. As expected, the induction of genes that are known to be YAP/TAZ targets, such as *CYR61*, *CTGF*, and *FOSB*, was largely dependent on YAP/TAZ (Fig. 6A). We then performed RNA-seq using YAP/TAZ KO and wild-type HEK293 cells treated with or without CCCP (20 μM, 3 h)(Fig. 6B and Supplementary Table 3). Intriguingly, genetic deletion of YAP/TAZ dramatically changed the expression pattern of the mitochondrial stress-responsive genes toward the pattern seen in untreated cells. In contrast, a relatively small portion of the genes was affected by CCCP treatment in KO cells (Fig. 6C). Further analysis showed that 62.8% of genes (574 of 914, consisting of 323 upregulated and 251 downregulated genes) required YAP/TAZ for proper induction in response to CCCP. Gene ontology analysis revealed that YAP/TAZ-dependent genes belonged to the ATF4-activated genes, TGFβ receptor signaling, PERK-regulated genes, and YAP/TAZ-stimulated genes (Fig. 6D). Among these genes, pathways involved in pro-inflammatory TNFα signaling, inflammation, and apoptosis were significantly enriched in GSEA (Fig. 6E). Additional analysis further confirmed the YAP/TAZ-dependent induction of genes with functions that are altered in liver injury, such as mitochondrial stress response and inflammation (Fig. 6F). These observations indicated a previously unrecognized profound role of YAP/TAZ in mitochondrial stress-induced transcriptomic alterations.

### Hepatocyte Yap/Taz promote liver injury and inflammation

**Fig. 7:**
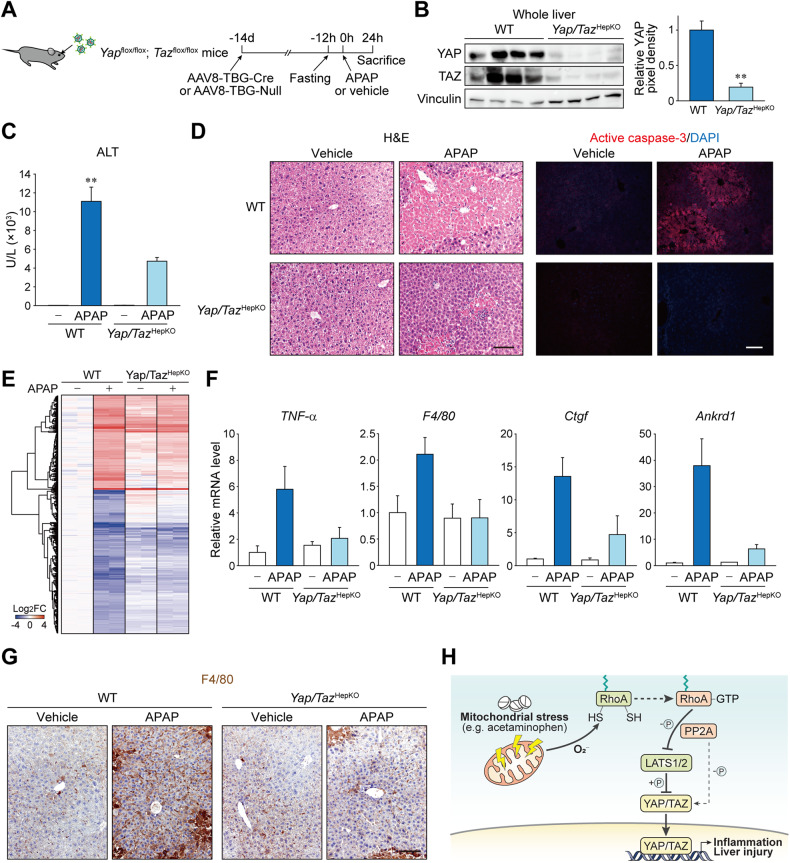
Hepatocyte YAP/TAZ promotes acetaminophen-induced liver injury and inflammation. **A**–**G** *Yap*/*Taz*^fl/fl^ mice were treated with AAV8-TBG-Cre or AAV8-TBG-Null control virus to induce hepatocyte-specific KO. After 14 days the mice were fasted for 12 h, orally given APAP or vehicle, then sacrificed 24 h later. **A** Experimental scheme. **B** Immunoblot for YAP/TAZ in mice with Cre virus (*Yap*/*Taz*^HepKO^) or control virus (WT). **C** Serum ALT. **D** Liver sections stained with H&E and fluorescently labeled with active caspase-3 antibody. Scale bars, 100 μm. **E** Heatmap showing APAP-induced genes from an RNA sequencing using the livers from mice. **F** qRT-PCR analysis in mouse liver. **G** Immunohistochemistry using F4/80 antibody. Wild-type samples were shared with Fig. 1D. Scale bar: 100 μm. **H** A proposed model for YAP/TAZ activation during drug-induced liver injury. For **B**, **C**, **F**, data represent the mean ± SEM. **P < 0.01, by Student’s *t-*test.

To verify the function of YAP/TAZ in the disease associated with mitochondrial stress in vivo, *Yap*/*Taz*^fl/fl^ mice were injected with AAV8-TBG-Cre to specifically deplete endogenous Yap/Taz in hepatocytes (Fig. 7A, B). The mice with hepatocyte-specific *Yap*/*Taz* KO (*Yap*/*Taz*^HepKO^) and control littermates injected with AAV8-TBG-Null (i.e., wild-type) were then subjected to a single APAP overdose. Hepatic GSH levels and APAP-protein adducts accumulation were comparable in Yap/Taz KO mice when compared to wild-type animals (Supplementary Fig. 6A, B). Moreover, Yap/Taz KO mice had unaltered *Cyp2e1* expression (Supplementary Fig. 6C). Nonetheless, we observed that serum ALT levels were significantly lower in *Yap*/*Taz*^HepKO^ mice when compared to wild-type mice (Fig. 7C). Consistently, histological assessments showed a marked reduction in APAP-induced hepatocyte death in *Yap*/*Taz*^HepKO^ mice (Fig. 7D). RNA-seq from the livers showed that APAP-induced transcription was significantly blunted in the absence of Yap/Taz in hepatocytes, similar to what has been observed in the CCCP-treated cell model (Fig. 7E and Supplementary Table 4). Of note, induction of *TNFα* and macrophage marker *F4/80* upon APAP overdose was significantly repressed in *Yap*/*Taz*^HepKO^ mice, implying the critical role of YAP/TAZ in promoting secondary inflammation after hepatocyte damage (Fig. 7F). Furthermore, the blockade of APAP-induced inflammation and macrophage proliferation in *Yap*/*Taz*^HepKO^ mice was verified using F4/80 immunostaining (Fig. 7G).

JNK signaling cascade is one of the major pathways causing APAP-induced toxicity in hepatocytes [27–30]. We found that phosphorylation of JNK1/2 was decreased in Yap/Taz KO mice (Supplementary Fig. 6D), which was in line with the previous report in endothelial cells showing that YAP/TAZ promotes the JNK pathway [31]. On the other hand, changes in hepatocyte markers and dedifferentiation were rather less in Yap/Taz KO mice compared to wild-type animals, indicating that the liver protection in Yap/Taz KO mice is not due to accelerated hepatocyte dedifferentiation (Supplementary Fig. 6E). Collectively, our data support the conclusion that mitochondrial stress induced by APAP overdose activates YAP/TAZ through superoxide-triggered RhoA signaling, which promotes inflammation and exacerbates liver injury (Fig. 7H).

## Discussion

YAP/TAZ has gained considerable attention because of the dramatic overgrowth of the liver following its overexpression [32]. Since then, they have been extensively studied as central players in cell growth and organ size control. Although their functional role in liver cell fate has recently been implicated, little is known regarding the upstream input to modulate YAP/TAZ activity in hepatocytes. In this study, we observed that YAP activity was substantially enhanced in hepatocyte damage, and was associated with mitochondrial stress and inflammation. Our data showed that mitochondrial stress promoted YAP/TAZ dephosphorylation, their nuclear accumulation, and target gene transcription. This was a result of RhoA oxidation by mitochondrial superoxide which resulted LATS-dependent and -independent activation of YAP/TAZ via PP2A. APAP-induced liver damage and macrophage recruitment were suppressed in hepatocyte-specific *Yap*/*Taz* knockout mice, suggesting that YAP/TAZ relay mitochondrial dysfunction leading to hepatocyte death and liver inflammation.

A number of cellular stresses, such as endoplasmic reticulum stress, hypoglycemic stress, and osmotic stress have been linked to the Hippo pathway [21]. We have newly identified that disruption of mitochondrial homeostasis is a disease-linked trigger for YAP/TAZ activation. This also suggests a previously unappreciated role of YAP/TAZ as players in mitochondrial retrograde signaling. In *C. elegans*, ATFS-1 has been well documented as the nuclear factor predominantly responsible for mitochondrial retrograde responses [33]. On the other hand, mammalian cells seem to have multiple transcription factors, which may enable wider range of cellular responses with a context-specific manner. For instance, ATF4 has been shown to be a key transcription factor for ISR signaling generated by the accumulation of unfolded proteins inside mitochondria [17, 18, 34]. Nonetheless, our observations showed that YAP/TAZ is an independent retrograde signaling nexus. Notably, it was recently demonstrated that YAP/TAZ cooperates with ATF4 to facilitate sorafenib-induced ferroptosis [35]. Our results support this idea since ATF4 target genes were enriched among the YAP/TAZ-dependent mitochondrial stress transcripts. These findings warrant further studies to determine whether YAP/TAZ activation is a prerequisite for IST and other retrograde signaling pathways.

Another important implication of our study is that mitochondrial stress-induced YAP/TAZ activation may play a role in homeostatic regulation of hepatocyte proliferation. Although YAP is mostly found in bile duct cells in the normal liver [36], our results showed that acute injury can dramatically increase active YAP levels in hepatocytes, particularly at the perimeter of the necrotic (i.e., proliferative) region. Consistently, early regeneration of hepatocytes following partial hepatectomy was previously found to be associated with high levels of YAP activity in humans and mice [37, 38], which was required for prompt liver regeneration [39]. Coincidentally, mitochondrial superoxide production is induced shortly after partial hepatectomy, as evidenced by an increase in malondialdehyde and oxidized proteins in mitochondria [40]. Mitochondrial stress may be a possible driver of YAP/TAZ activation observed in regenerating hepatocytes after injury or surgical resection. Our results also showed that YAP/TAZ mediate the inflammatory response following APAP intoxication. Considering that a mild inflammatory response facilitates regeneration after partial hepatectomy [41], activation of YAP/TAZ by mitochondrial stress may support liver regeneration through intercellular crosstalk between hepatocytes and non-parenchymal cells.

The newly discovered signaling mechanism in this study also raises a fundamental question regarding the potential benefits that cells may receive from activating YAP/TAZ in response to mitochondrial superoxide. High concentrations of ROS irreversibly destroy cellular structures and, resulting tissue injury. In contrast, low concentrations are required for homeostasis by triggering defense mechanisms that prevent cellular damage from other stresses, which is termed as mitohormesis [42, 43]. Given that hepatocytes are the primary site of xenobiotics metabolism, they are frequently exposed to oxidative stress and mitochondrial damage. Based on the current findings, it may be possible that YAP/TAZ are transiently activated by sublethal stress for mitohormetic adaptation, while mostly remaining inactive under physiological conditions.

Considering the previously known regenerative functions of YAP/TAZ in the liver and our new findings showing a pro-inflammatory role, we speculate that the YAP/TAZ promote proliferation under conditions involving massive hepatocyte loss such as partial hepatectomy but induce inflammation in acute phase of liver injury. Nonetheless, future studies are yet needed to investigate by which associated factor YAP/TAZ exert opposing roles in hepatocytes.

## Methods

### In vivo experiments

Yap^flox/flox^ (#027929; The Jackson Laboratory, Maine) and Wwtr1^flox/flox^ (#032669; The Jackson Laboratory) were originally established by Dr. Eric Olson (University of Texas, Southwestern). The mice were bred to yield Yap^flox/flox^; Wwtr1^flox/flox^. For hepatocyte-specific Yap/Taz knockout (Yap/Taz^HepKO^), male mice of 7- or 8 weeks old age were intravenously administered with AAV8-TBG-Cre virus, 2 weeks before experiment. Control mice were given AAV8-TBG-Null virus. Mice were fasted for 12 h, given APAP (300 mg/kg) intraperitoneally, and sacrificed after 6-24 h, as indicated. Sample sizes were kept minimal based on previous experiences without statistical methods and animals are randomly allocated to experimental groups with no blinding. Mice were bred and maintained under specific pathogen-free conditions with a 12-hour dark/12-hour light cycle and controlled temperature and humidity. Animal studies were approved by the Institutional Animal Care and Use Committee of Seoul National University and were conducted according to guidelines. Human tissue slides were obtained from Biochain.

### Cell culture and treatment

Primary hepatocytes were isolated from the liver of 8-week-old male mice by standard two-step collagenase perfusion and centrifugation. Isolated hepatocytes were maintained in William’s E media (#LM017-02; Welgene, Korea) on a collagen-coated plate (#354400; Corning, New York). AML12 cells were cultured in DMEM/F12 medium (#LM002-04; Welgene) with 10% FBS (#F2442; Sigma, Missouri) and 1% penicillin/streptomycin (#LS202-02; Welgene), supplemented with insulin (10 μg/ml), transferrin (5.5 μg/ml), selenium (5 ng/ml), and dexamethasone (40 ng/ml). HEK293, MIHA, and Huh7 cells were cultured in DMEM (#LM001-05; Welgene) containing 10% FBS and 1% penicillin/streptomycin. All cells were cultured at 37°C within a humidified chamber with 5% CO2.

YAP inhibitory signals were introduced as following: Serum starvation (16 h); high density (100% confluence, 24 h); sorbitol (0.5 M, 1 h); cytochalasin D (1 μM, 1 h); latrunculin B (0.25 mg/ml, 1 h); and 2-DG (2-deoxyglucose; 25 mM, 1 h). LB-100 was pretreated in LATS1/2 KO cells at a concentration of 5 μM for 1 h and in wild-type cells at a concentration of 10 μM for 1 h.

### Gene editing

pSpCas9(BB)-2A-Puro (#62988; Addgene, Massachusetts) and lentiCRISPRv2 (#52961; Addgene) were used for CRISPR-guided gene knockout and knock-in. Briefly, guide RNAs were cloned into the empty vector. Cells were then transfected with plasmids, selected for antibiotics resistance, and FACS-sorted for single cells. Expanded single clones were screened by protein immunoblotting and/or genomic DNA sequencing. For knock-in, single-stranded oligodeoxynucleotides containing mutagenic sequence were co-transfected. For CRISPR-guided gene silencing, lenti_dCas9-KRAB-MeCP2 (#122205; Addgene) was used. The sequences for guide RNAs and oligonucleotides used for gene editing are listed in the Supplementary Table 5.

### RNA isolation and quantitative PCR

Total RNA was isolated using RNeasy Mini kit (Qiagen, Germany) for cell lines or TRIzol reagent (Invitrogen, Massachusetts) for mouse livers. Reverse transcription was done using AccuPower RT premix (Bioneer, Korea). qRT-PCR was performed using AccuPower 2X GreenStar qPCR Master Mix (Bioneer) using the CFX Connect Real-Time PCR detection system (Bio-Rad, California). After polymerase chain reaction amplification, the melt curve of each amplicon was determined to verify its accuracy. All mRNA levels were normalized to expression of housekeeping genes. Primer sequences used for PCR are available in Supplementary Table 5.

### Western blot

Gels containing Phos-tag (Wako, Japan) were prepared according to the manufacturer’s instructions. Cell or tissue lysates were quantified, separated by SDS-PAGE and transferred to nitrocellulose membranes (GE Healthcare, Illinois) followed by immunoblotting. For immunoprecipitation, cells were lysed with buffer containing 150 mM NaCl, 1 mM EDTA, 0.5% NP-40, and 10% Glycerol. The lysates were pre-cleaned by centrifugation and incubation with Protein A beads (#L00273, Genscript, New Jersey). Then, the lysates were incubated with GTP-RhoA antibody and Protein A beads. The immunoprecipitated proteins were eluted by boiling beads in a denaturing buffer and subjected to immunoblotting. For immunoblotting, proteins of interest were probed with primary antibodies and horse-radish peroxidase-linked secondary antibodies for chemiluminescence detection. Antibodies used for immunoblottings are listed on the Supplementary Table 6. All uncropped immunoblots are provided as Supplementary material.

### Immunofluorescence analysis

Cells were seeded on coverslips pretreated with poly-L-ornithine (#P3655; Sigma). Cells were fixed in 4% paraformaldehyde for 15 min and permeabilized by 0.1% Triton X-100. The cells were then stained with YAP/TAZ antibody and DAPI. Images were captured with TCS SP8 confocal microscope (Leica, Germany).

### Transmission electron microscopy

Mice liver section was fixed in Karnovsky’s fixative immediately after harvest. Samples were then post‐fixed by 1% osmium tetraoxide. After overnight incubation with 0.5% uranyl acetate, the samples were dehydrated and polymerized in Spurr’s resin. The images were taken using a LIBRA 120 transmission electron microscope (Zeiss, Germany).

### RNA sequencing and bioinformatic analysis

YAP/TAZ KO and wild-type HEK293 cells treated with 20 μM of CCCP for 3 h (*N* = 4) were used for RNA sequencing. Liver tissues from Yap/Taz^HepKO^ and wild-type mice treated with or without 300 mg/kg acetaminophen for 24 h (*N* = 8) were also used. RNA was prepared according to the manufacturer’s protocol using a Qiagen RNeasy Mini kit. cDNA synthesis was performed using dUTP and sequenced on the Illumina platform. Expression levels for each gene were identified using FPKM. Differentially expressed genes (DEGs) were identified with DESeq2. RNA-seq data from Gene Expression Omnibus were retrieved and reanalyzed in the study (GSE136103, GSE166868, GSE167033). For heatmap visualization and categorization of DEGs, only genes with statistical significance (*p* < 0.01) were analyzed (Supplementary Tables 3–6). GOBP (ROS signaling, mitochondrial membrane depolarization, and mitophagy), MSigDB Hallmark gene set (macrophage activation), and Cordenonsi YAP conserved signature gene sets were used for grouping DEGs. To identify YAP/TAZ-dependent genes, DEGs between YAP/TAZ KO groups were ruled out from DEGs between wild-type groups. Gene ontology enrichment analysis of DEGs and KEGG pathway analysis were done by DAVID functional annotation tool. Gene set enrichment analysis was done using MSigDB Hallmark gene sets or Cordenonsi YAP conserved signature gene set.

### Flow cytometry

Mitochondrial superoxide levels were indicated by staining cells with either MitoNeoD (5 μM) [44] or MitoSOX Red (2 μM) for 30 min at 37°C in the culture vessels. SpectraMAX iD3 (Molecular Devices, California) was used with 544 nm excitation and 605 nm emission wavelengths to detect MitoNeoD fluorescence. FACS Calibur cytometer (BD, New Jersey) were used for flow cytometric analysis of MitoSOX intensity and 10,000 gated events were collected for each sample.

### Glutathione quantification

Glutathione levels in the protein-precipitated liver lysates were measured according to manufacturer’s instructions using a GSH assay kit (Biomax, Korea)

### Statistical analysis

All statistical analysis was performed using SigmaPlot. ImageJ was used for densitometry analysis of western blot. Criteria for statistical significance were considered to be significant when *p < 0.05, and **p < 0.01 from Student’s *t*-test.

### Supplementary information

Supplementary Fig.s

Supplementary Table 1

Supplementary Table 2

Supplementary Table 3

Supplementary Table 4

Supplementary Table 5

Supplementary Table 6

Uncropped Western Blots

Reproducibility checklist

## Data Availability

All datasets generated and analyzed during this study are included in this published article and its Supplementary Information files. Additional data are available from the corresponding author on request.
