# Supplementary material for: Mitochondrial stress activates YAP/TAZ through RhoA oxidation to promote liver injury
Source: Cell Death Dis. 2024 Jan 15;15(1):51. doi: 10.1038/s41419-024-06448-5 (PMC10789791; doi:10.1038/s41419-024-06448-5)
Supplement: Supplementary file 1 — Supplementary Fig.s [file 41419_2024_6448_MOESM1_ESM.pdf]

Supplementary Figure legends

Supplementary Figure 1. Acetaminophen causes mitochondrial damage and YAP activation in hepatocytes

(**A**) Transcripts from cells of cirrhotic or healthy human livers with lineage were analyzed for YAP/TAZ and target gene expression. The data were extracted from GEO GSE136103 (n=5 each).

(**B**) Immunohistochemistry for active YAP in mouse liver after APAP intoxication. Mice are starved overnight and treated with acetaminophen (300 mg/kg) or vehicle for 24 h.

(**C**) APAP injection causes severe mitochondrial damage in hepatocytes. Transmission electron micrograph of mouse liver, 24 h after APAP injection. Scale bar: 1 μm.

(**D**) Temporal pattern of mitochondrial damage and YAP activation by APAP. Transmission electron micrograph and immunohistochemical image for active YAP were taken from mouse liver. Mice are starved overnight, treated with acetaminophen (300mg/kg), and sacrificed after 6h or 12h. Scale bars: 1 μm (*left*); 100 μm (*right*).

Supplementary Figure 2. YAP/TAZ are activated by mitochondrial stress

(**A**) TAZ is dephosphorylated by mitochondrial stress. Cells were treated with increasing concentrations of CCCP (10, 20, 50 μM)

(**B**) Mitochondrial stress induces TAZ nuclear localization. HEK293 cells were trated with CCCP (50 μM) for 1 h and then stained for immunofluorescence. Scale bar: 50 μm.

(**C**) Mitochondrial stress activates YAP in hepatic non-parenchymal cells. *Left*, Hepatic stellate cells and Kupffer cells are isolated from mice were subjected to mitochondrial stress by CCCP for indicated times. *Right*, validation of cell purity by immunostaining for marker proteins. Scale bar: 50 μm.

(**D**) Mitochondrial stress overrides other Hippo activating signals to induce YAP phosphorylation. HEK293 cells were pre-treated with TAZ inhibitory signals (see *Methods*), then treated with CCCP. Nuclear localization was visualized by fluorescently labeled immunostaining for TAZ. Scale bar: 50 μm.

(**E**) Phos-tag immunobloting against TAZ after treatment with MitoPQ (10 μM) for indicated times.

(**F**) After 1h of serum starvation, cells were treated with mitoTempol (100 μM) for 1h, then treated with CCCP (50 μM) and analyzed using Phos-tag immunoblotting.

(**G**) Phos-tag immunoblotting for TAZ. Cells were transfected with HA-tagged SOD2 expression vector and treated with CCCP for 1 h after 24 h.

Supplementary Figure 3. RhoA recognizes superoxide to activate YAP

(**A**) Validation of homozygous CRISPR-guided knock-in for RhoA C16A/C20A HEK293 cells. Sanger sequencing was done using genomic DNAs from a monoclonal knock-in cell line.

(**B**) Hydrogen peroxide does not activate YAP. Immunoblot using Phos-tag gels of YAP activity in HEK293 cells treated with indicated concentrations of hydrogen peroxide or 50 μM CCCP for 1 h. The cells were serum-starved 1 h prior to experiment.

Supplementary Figure 4. YAP dephosphorylation by CCCP is not from integrated stress response, mitophagy, mitochondrial proteostasis or ferroptosis

(**A**) Changes in expression of marker proteins for mitochondrial dysfunction. HEK293 cells were treated with CCCP (20 and 50 μM) for 1 h.

(**B**) *Left*, Wild type, eIF2α S51A knock-in, ATF4 KO HEK293 cells treated with CCCP (50 μM) analyzed by immunoblotting using Phos-tag gels. *Right*, sequence confirmation of the targeted mutation using genomic DNA of EIF2AS1 S51A knock-in cells by Sanger sequencing.

(**C-F**) Phos-tag immunoblottings for YAP. (**C**) HEK293 cells were treated with ISRIB (200 nM) and treated with CCCP (50 μM, 1 h). (**D**) HEK293 cells were treated with Mdivi-1 (10 μM) prior to CCCP treatment. (**E**) HEK293 cells were treated with CCCP (50 μM) or MitoBlock-6 (10 μM) for indicated times. (**F**) HEK293 cells were treated with ferrostatin-1 (5 μM) for 1h, then treated with CCCP (50 μM) for 1 h.

For **A-F**, HEK293 cells were serum-starved 1 h prior to experiment.

Supplementary Figure 5. Antioxidants do not promote YAP phosphorylation at the basal level

Immunoblotting for YAP was done using HEK293 cells treated with N-acetylcysteine (NAC; 2 mM) and mitoTempol (100 μM) for 1h.

Supplementary Figure 6. Yap/Taz mediate JNK1/2 phosphorylation, but not bioactivation of APAP or hepatocyte dedifferentiation

(**A-E**) *Yap*/*Taz*$^{fl/fl}$ mice were treated with AAV8-TBG-Cre or AAV8-TBG-Null control virus to induce hepatocyte-specific KO. After 14 days the mice were fasted for 12 h, orally given APAP or vehicle, then sacrificed 24 h later. (**A**) Hepatic GSH level was examined using whole liver lysate. (**B**) NAPQI-protein adducts were immunostained in the liver using anti-acetaminophen antibody. Scale bar: 100 μm. (**C**) Cyp2e1 expression from liver RNA-sequencing data are shown. (**D**) Hepatic protein expression for JNK/MAPK signaling. (**E**) Heatmap showing markers for hepatocyte dedifferentiation form RNA-sequencing data. Log2 fold change of DEGs with statistical significance in APAP-treated wild-type mice compared to the vehicle-treated wild-type mice are displayed.

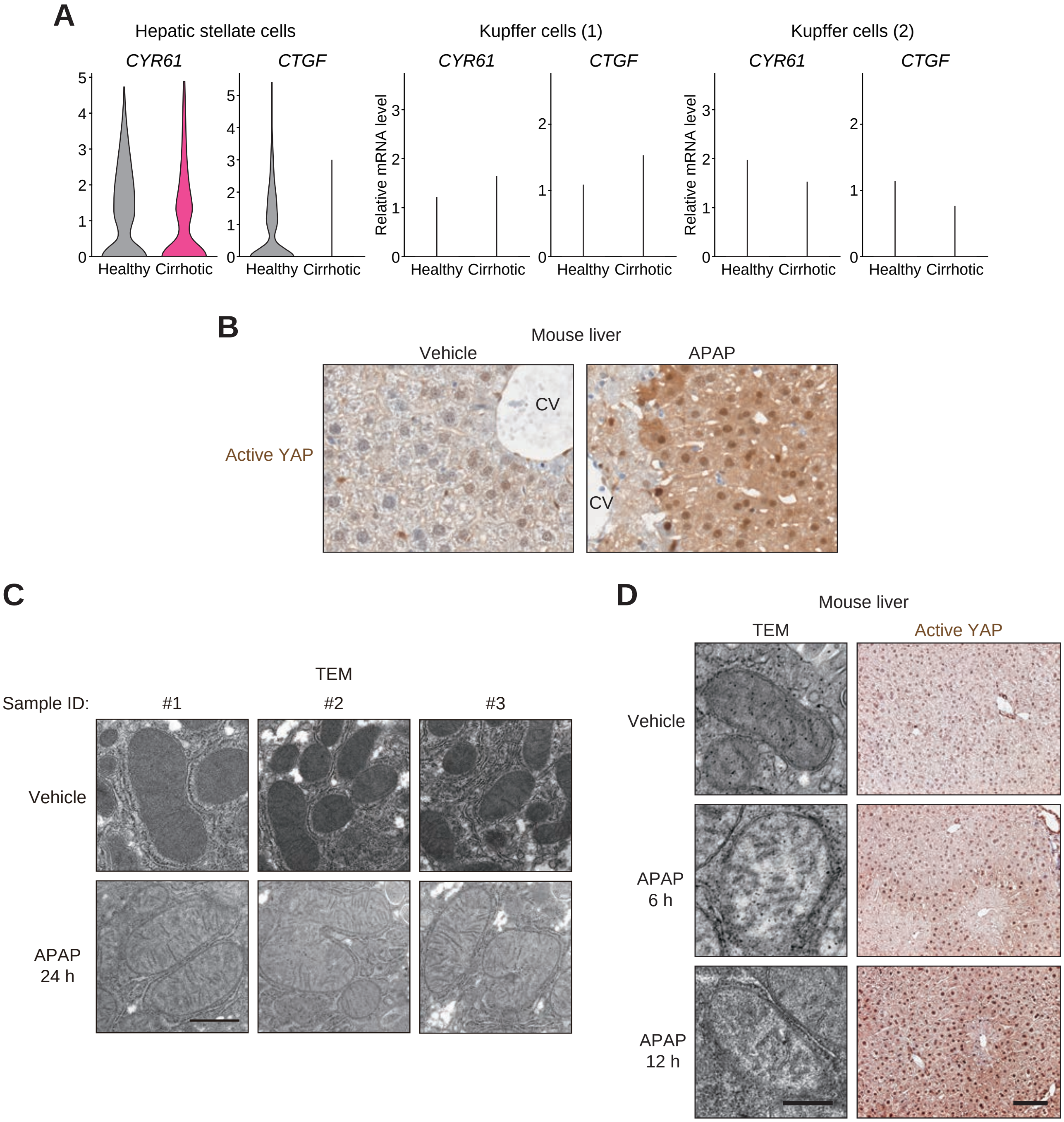

Supplementary Figure 1

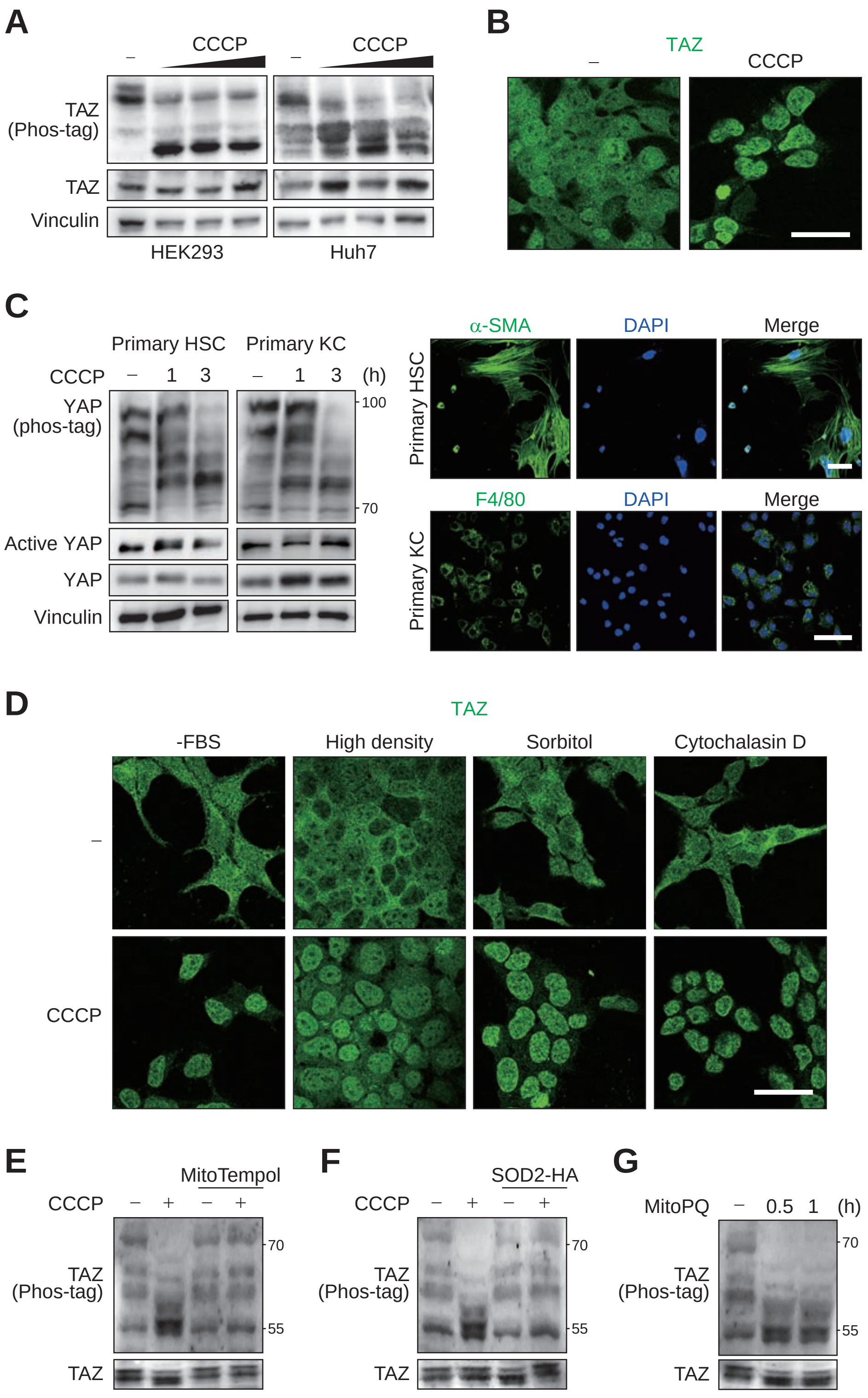

Supplementary Figure 2

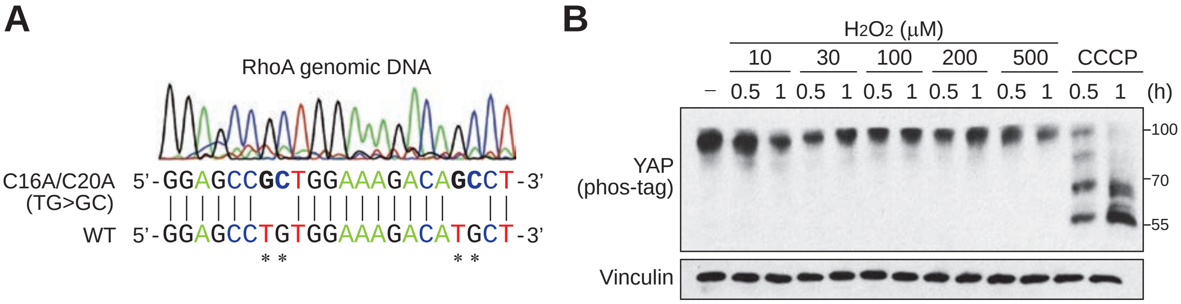

Supplementary Figure 3

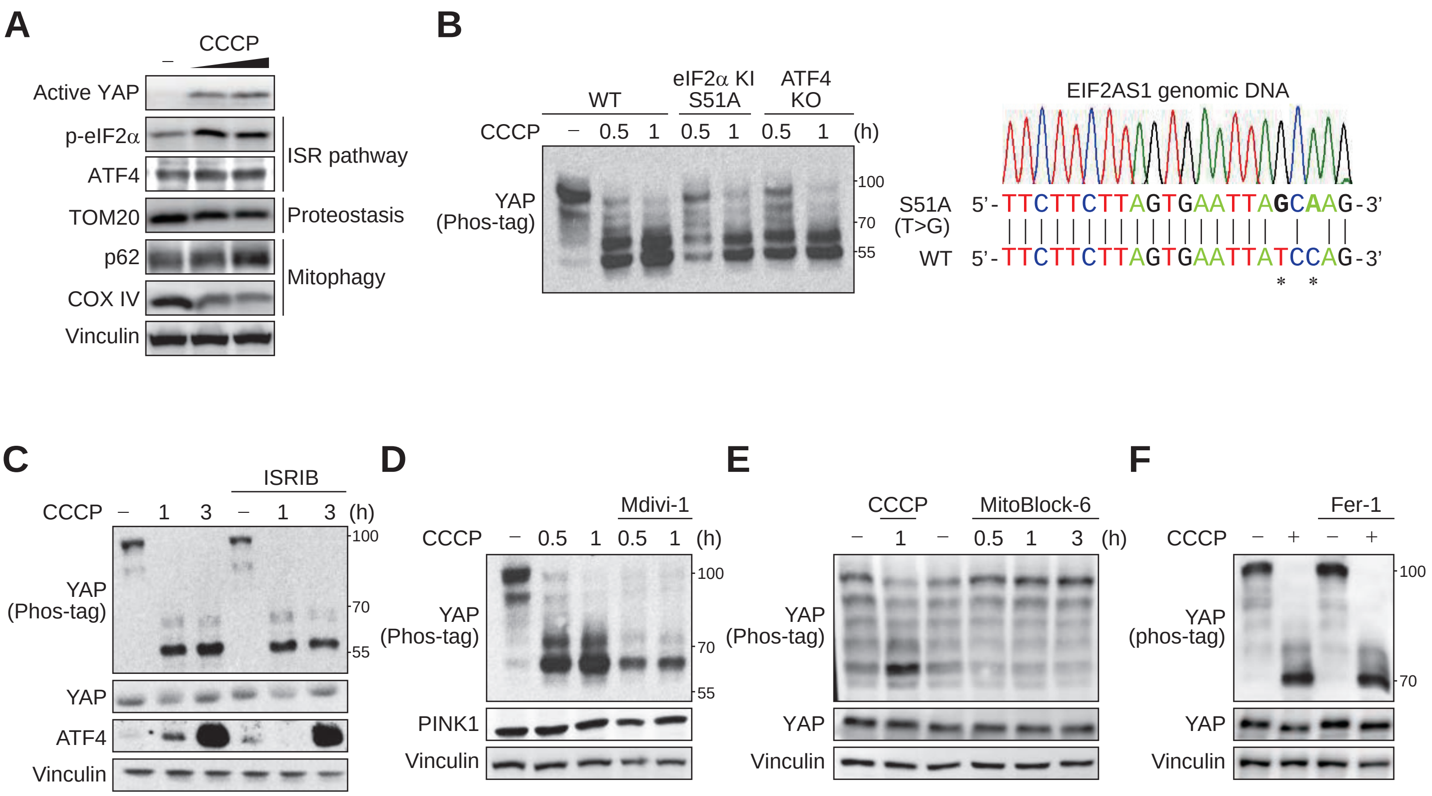

Supplementary Figure 4

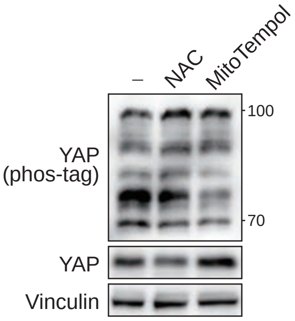

Supplementary Figure 5

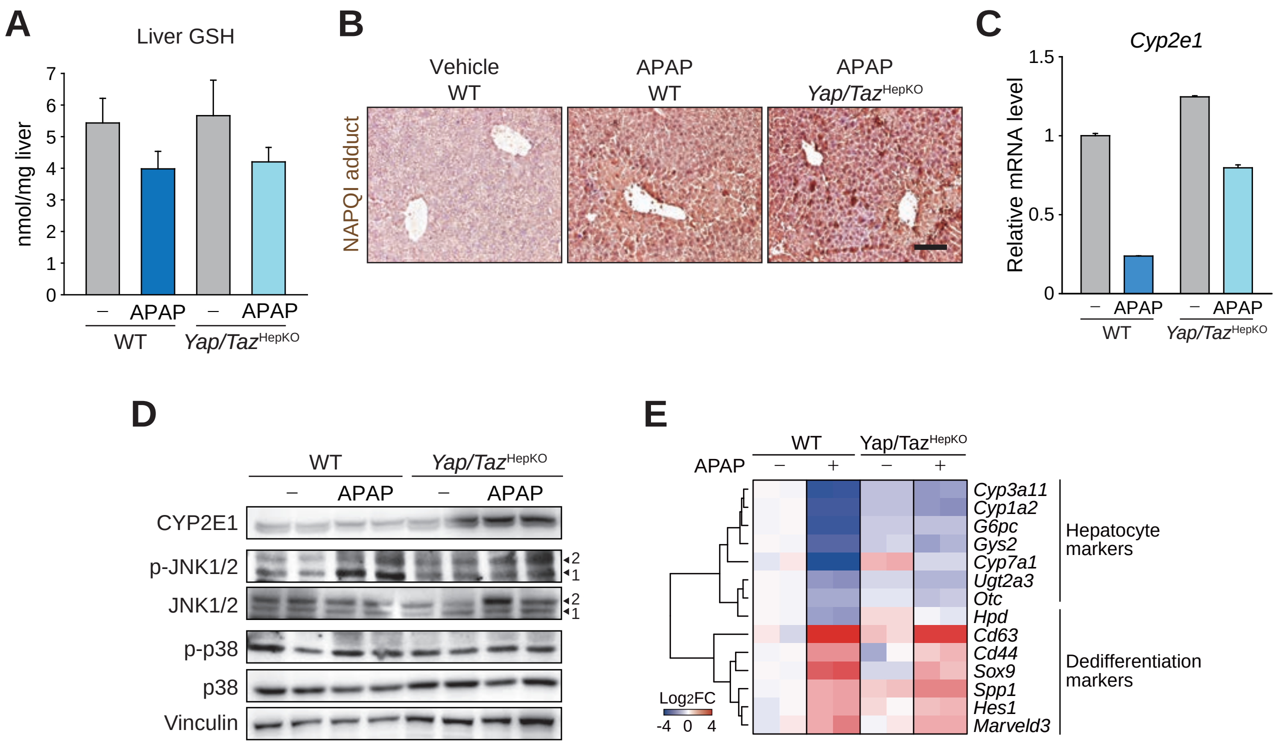

Supplementary Figure 6
