# Supplementary material for: Mitochondrial stress activates YAP/TAZ through RhoA oxidation to promote liver injury
Source: Cell Death Dis. 2024 Jan 15;15(1):51. doi: 10.1038/s41419-024-06448-5 (PMC10789791; doi:10.1038/s41419-024-06448-5)

Uncropped western blots

Figure 1E

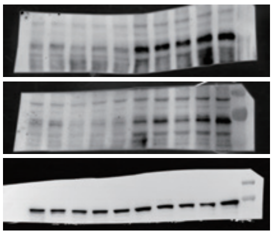

Active YAP

YAP

Vinculin

Figure 1F

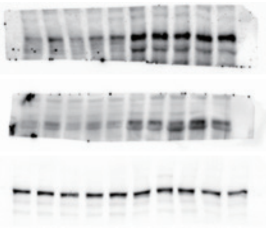

Active YAP

YAP

Vinculin

Figure 1G

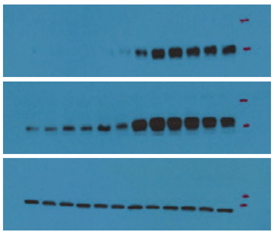

Active YAP

YAP

Vinculin

Figure 2D

Huh7 HEK293

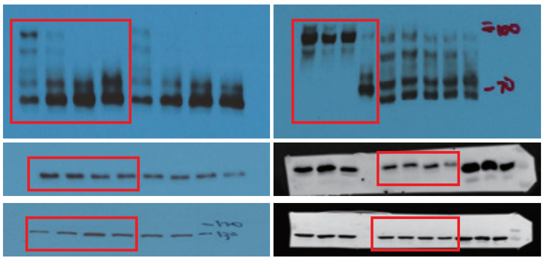

YAP (Phos-tag)

YAP

Vinculin

Figure 2E

Huh7 AML12 HEK293

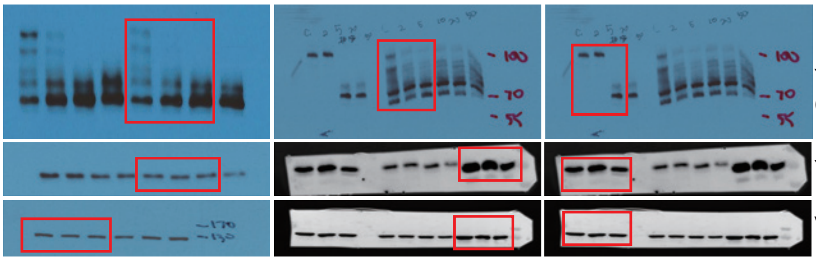

YAP (Phos-tag)

YAP

Vinculin

Figure 2F

Huh7 HEK293

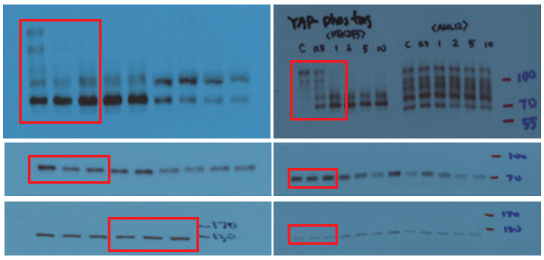

YAP (Phos-tag)

YAP

Vinculin

Figure 2G

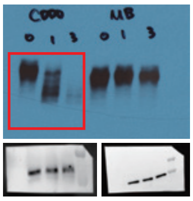

YAP (Phos-tag)

YAP Vinculin

Figure 2H

MIHA

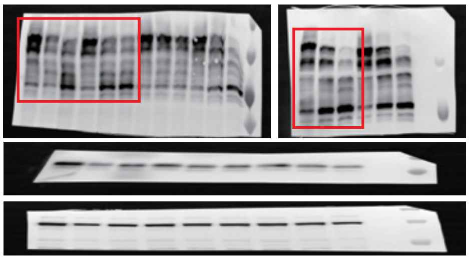

YAP (Phos-tag)

YAP

Vinculin

Figure 2H

Primary hepatocyte

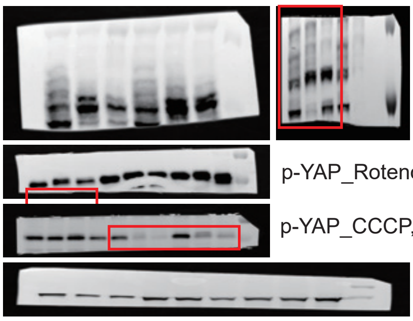

YAP (Phos-tag)

p-YAP_Rotenone

p-YAP_CCCP,FCCP

Vinculin

Figure 2K

-FBS 2-DG Sorbitol

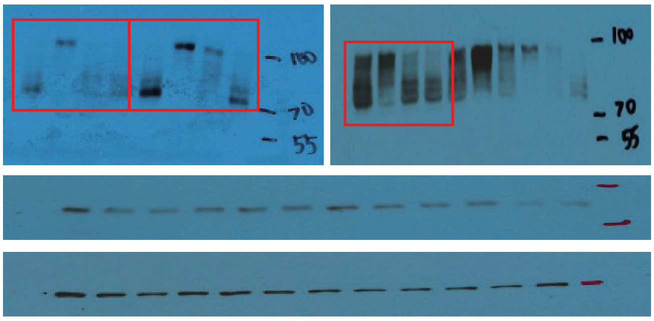

YAP (Phos-tag)

YAP

Vinculin

Figure 2K

High density Cytochalasin D Latrunculin B

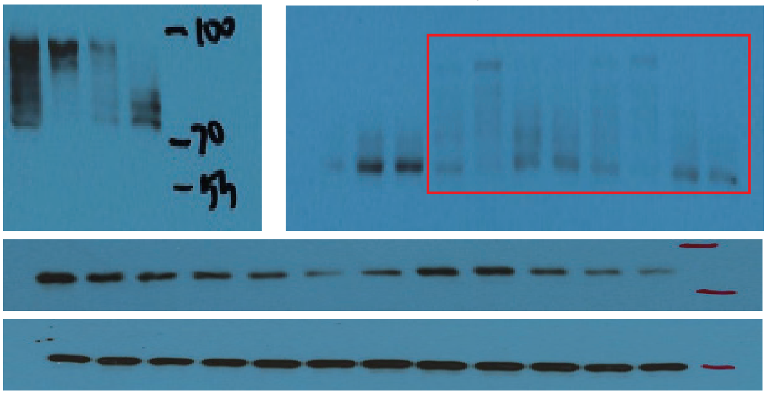

YAP (Phos-tag)

YAP

Vinculin

Figure 3B

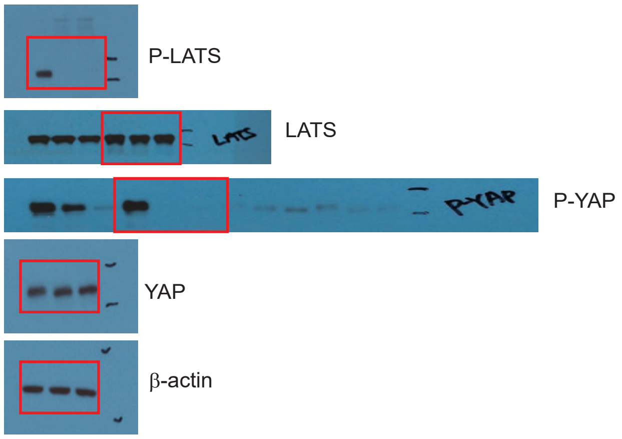

Figure 3C

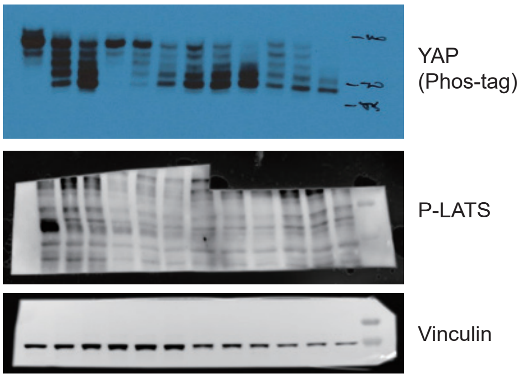

Figure 3D

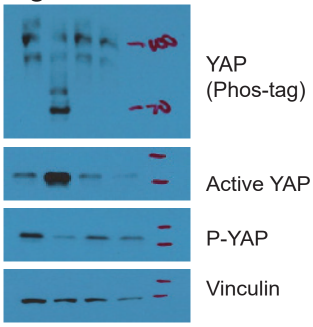

Figure 3G

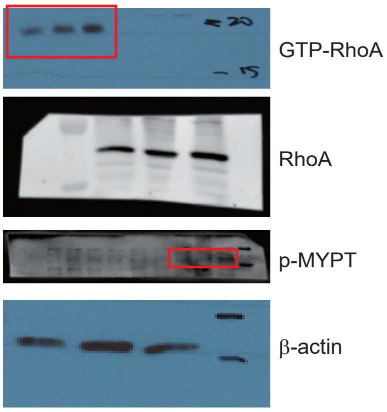

Figure 4A

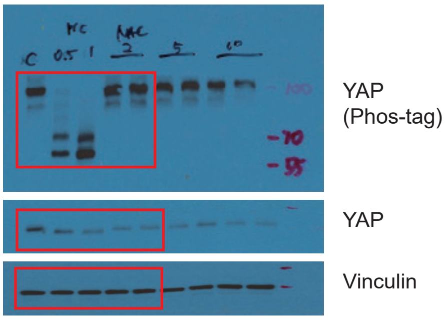

Figure 4B

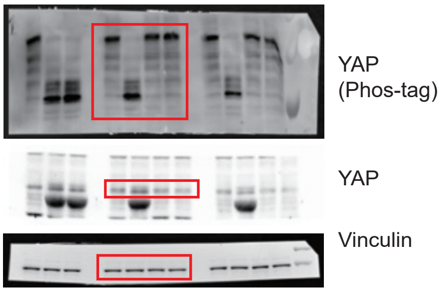

Figure 4F

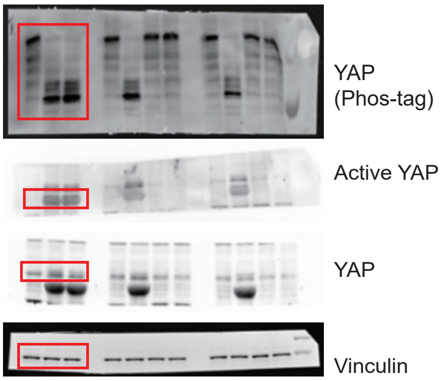

Figure 4H

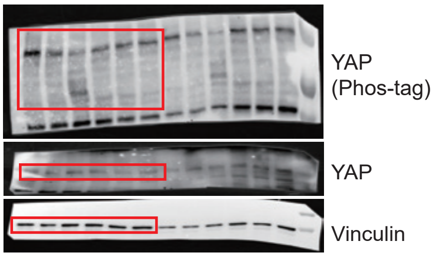

Figure 4C

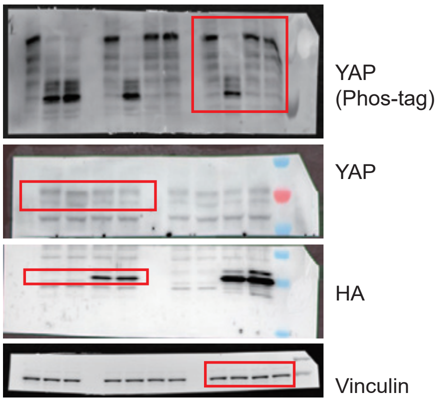

Figure 5B

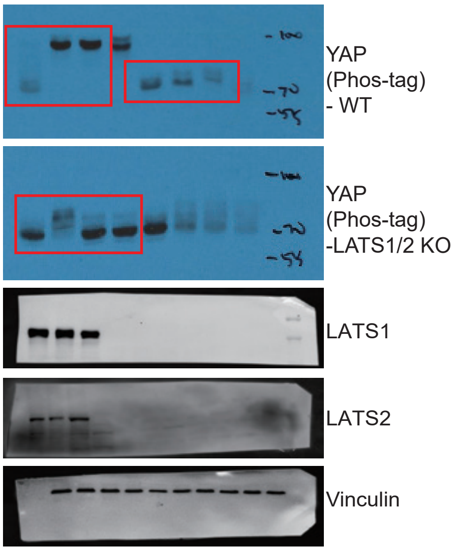

Figure 5A

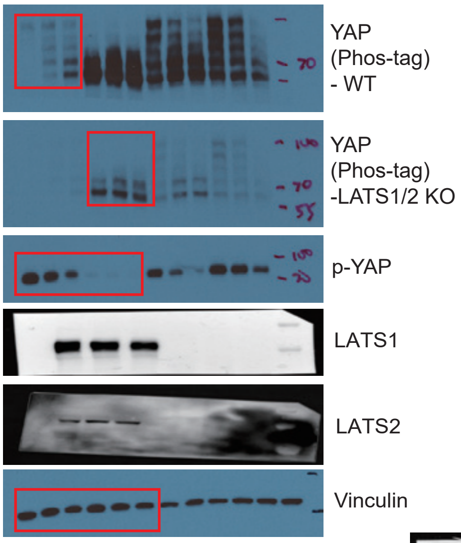

Figure 4K

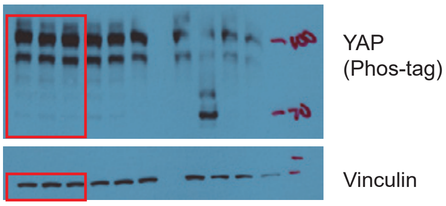

Figure 5C

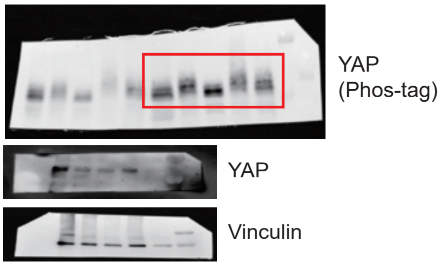

Figure 5D

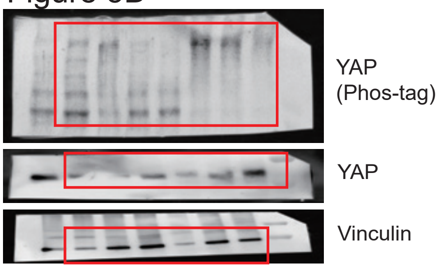

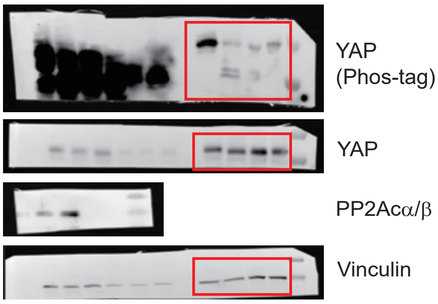

Figure 6A

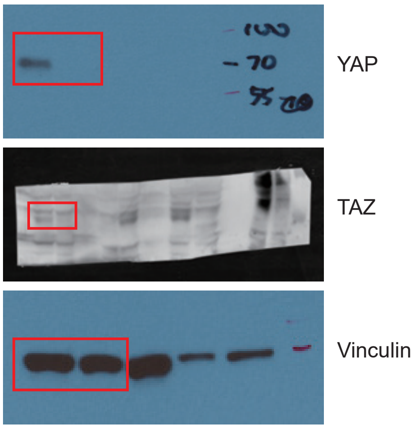

Figure 7B

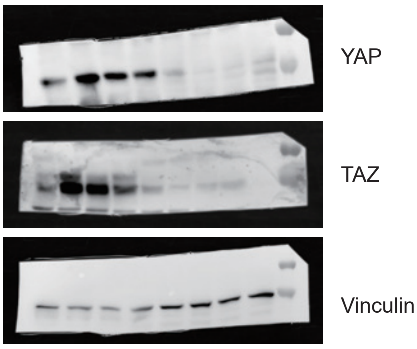

Supplementary Figure 2A

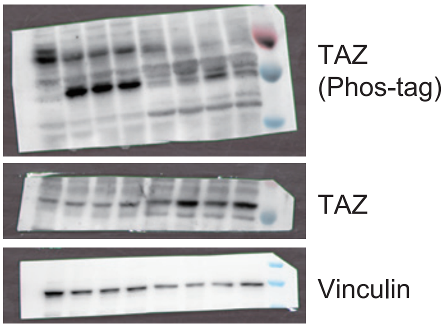

Supplementary Figure 2C

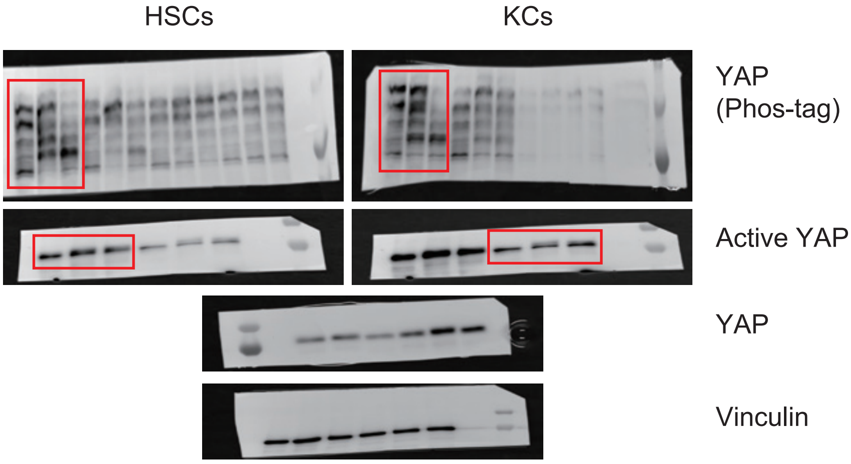

Supplementary Figure 2E

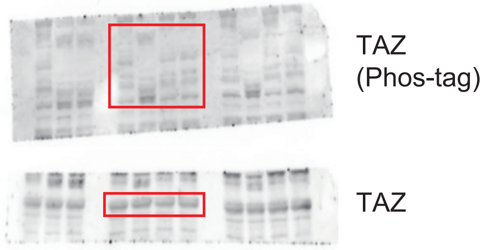

Supplementary Figure 2F

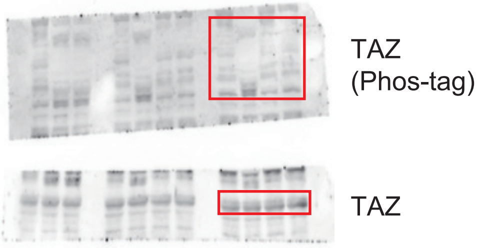

Supplementary Figure 2G

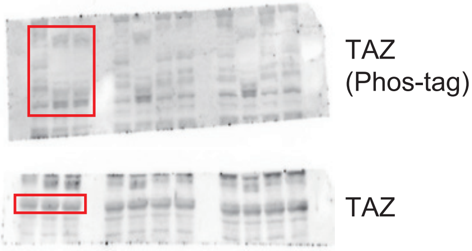

Supplementary Figure 3B

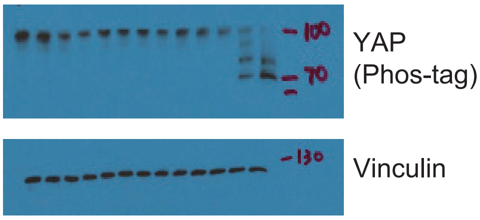

Supplementary Figure 4A

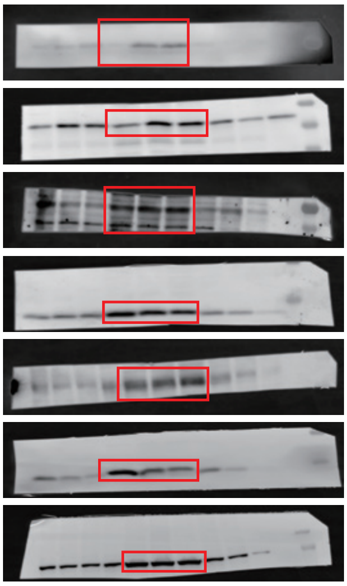

Supplementary Figure 4B

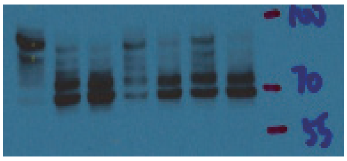

Supplementary Figure 4C

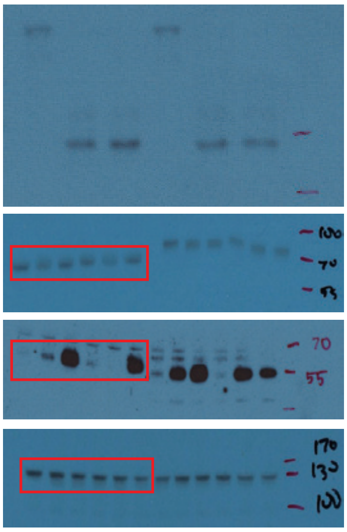

Supplementary Figure 4D

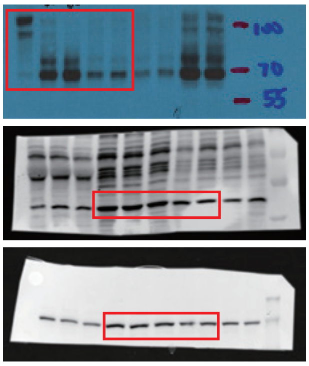

Supplementary Figure 4E

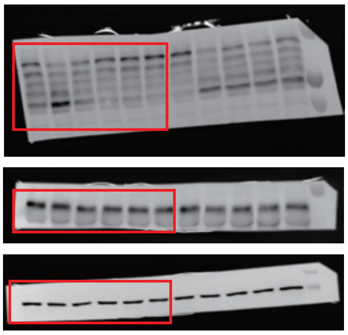

Supplementary Figure 4F

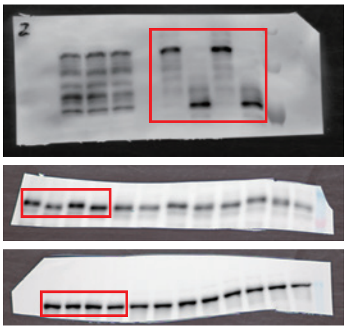

Supplementary Figure5

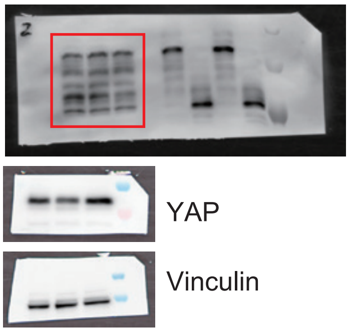

Supplementary Figure6D

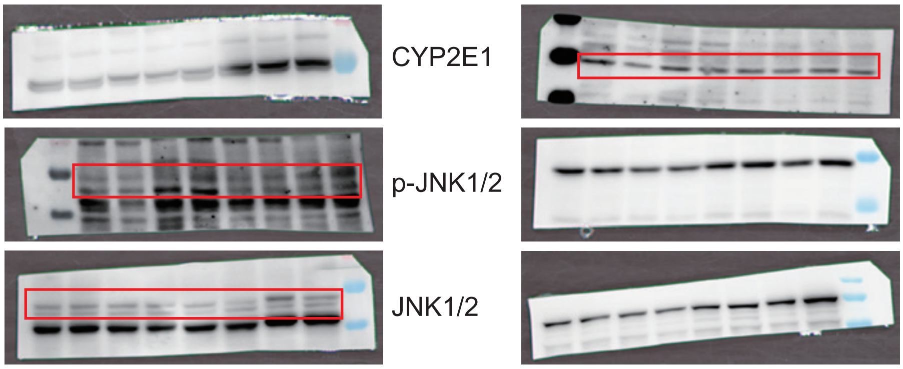

Supplement: Supplementary file 8 — Uncropped Western Blots [file 41419_2024_6448_MOESM8_ESM.pdf]
